# Machine learning-based protein crystal detection for monitoring of crystallization processes enabled with large-scale synthetic data sets of photorealistic images

**DOI:** 10.1007/s00216-022-04101-8

**Published:** 2022-06-04

**Authors:** Daniel Bischoff, Brigitte Walla, Dirk Weuster-Botz

**Affiliations:** Technical University of Munich, Institute of Biochemical Engineering, Boltzmannstr. 15, Bavaria 85748 Garching, Germany

**Keywords:** Protein crystallization, Automated image analysis, Synthetic data sets, Deep learning

## Abstract

Since preparative chromatography is a sustainability challenge due to large amounts of consumables used in downstream processing of biomolecules, protein crystallization offers a promising alternative as a purification method. While the limited crystallizability of proteins often restricts a broad application of crystallization as a purification method, advances in molecular biology, as well as computational methods are pushing the applicability towards integration in biotechnological downstream processes. However, in industrial and academic settings, monitoring protein crystallization processes non-invasively by microscopic photography and automated image evaluation remains a challenging problem. Recently, the identification of single crystal objects using deep learning has been the subject of increased attention for various model systems. However, the advancement of crystal detection using deep learning for biotechnological applications is limited: robust models obtained through supervised machine learning tasks require large-scale and high-quality data sets usually obtained in large projects through extensive manual labeling, an approach that is highly error-prone for dense systems of transparent crystals. For the first time, recent trends involving the use of synthetic data sets for supervised learning are transferred, thus generating photorealistic images of virtual protein crystals in suspension (PCS) through the use of ray tracing algorithms, accompanied by specialized data augmentations modelling experimental noise. Further, it is demonstrated that state-of-the-art models trained with the large-scale synthetic PCS data set outperform similar fine-tuned models based on the average precision metric on a validation data set, followed by experimental validation using high-resolution photomicrographs from stirred tank protein crystallization processes.

**Supplementary Information:**

The online version contains supplementary material available at 10.1007/s00216-022-04101-8.

## Introduction

Protein purification from impure bacterial cell lysate by crystallization is a promising alternative to diffusion-controlled preparative chromatography with potential economic benefits [1, 2] and improved sustainability aspects. While the limited crystallizability of proteins often restricts a broad application of crystallization as a purification method, advances in molecular biology, as well as computational methods are pushing the applicability towards integration in biotechnological downstream processes [3–6]. In industrial and academic settings, monitoring protein crystallization processes non-invasively by microscopic photography and automated image evaluation remains a challenging problem, while being essential for rapid development cycles and precise process control. Bright-field imaging is a general illumination technique for optical microscopy that can be used to monitor crystallization processes in static drops, or in situ, with the advantage of being non-invasive and cost-efficient [7, 8]. However, crystals may appear predominantly transparent, surrounded fully or partially by visible edges due to the system’s refractive properties and lighting conditions. Additionally, visual monitoring of general in situ crystallization processes may be subject to varying sources of noise and varying object sizes, resulting in (non-trivial) challenges for image analysis techniques that are further strained by the requirement for fast evaluation times to enable statistically informed feedback for process controls [9]. Early methods for crystal detection relied on a combination of image preprocessing steps, thresholding, edge detection and segmentation, followed by size-based or convexity-based particle sieving [10, 11]. Further developments included a multi-scale approach, in which edges were detected at two image scales and then combined for segmentation, resulting in binary images with connected components representing crystals [9]. However, segmentation-based approaches tended to merge overlapping or attached crystals and therefore distort the real crystal size distribution in dense systems. Further analysis and subsequent splitting of the object’s concave boundaries overcame this shortcoming, but was not able to separate attached crystals that form convex hulls and relied on high-quality images with clearly visible crystal borders [12, 13]. An alternative to the segmentation-based approach was introduced with the SHARC algorithm, which identified line segments and then combined them based on the observation that parallel line segments in spatial proximity probably represent either the same crystal edge interrupted by noise, or opposite crystal edges [14]. The M-SHARC algorithm improved upon this by fitting line segments to several predefined wire-frame models, thus achieving crystal outline detection without image segmentation. Nevertheless, the authors still detailed challenges with blurred or low-contrast crystal edges [15]. Additional work focused on the extraction of 3D shape descriptors from 2D images, or more elaborate imaging setups with less focus on the resistance to noise, requiring single- or multi-scale segmentation as one of the early image analysis steps [16–19]. In a parallel development fuelled by the automatization of protein crystallization experiments, machine learning methods began to be applied to classification tasks and accelerated the search for crystals suitable for X-Ray diffraction and structure determination [20]. While conventional machine learning approaches required careful engineering, the development of the deep learning subcategory enabled to learn relevant patterns automatically [21]. Further advances in object detection were applied to the task of detecting individual crystals of the $\upalpha$- and ${\upbeta }$-forms of l-glutamic acid by fine-tuning a two-stage Mask R-CNN detector model [22, 23], which was later used to estimate the crystals population growth rate [24]. While two-stage detectors are able to yield per-object masks, their disadvantages are slow inference times and low detection rates of crystals with high-aspect ratios in dense images, caused by non-maximum suppression (NMS) of non-rotated rectangular region proposals. For a high-throughput system, the mask prediction was omitted completely, the system instead focusing on the classification of morphology of individual crystals of the pharmaceutical ingredient indometacin [25]. The problem of slow inference times was addressed by the introduction of the focal loss for general object detection tasks, enabling fast one-stage detectors that competed in accuracy with state-of-the-art detectors [26]. In the context of crystal detection, one-stage detectors were first applied to a model system of low-aspect ratio crystalline sodium chloride [27]. More recently, a model crystallization system of an aqueous taurine solution was extensively studied using a one-stage state-of-the-art S$^{2}$A-Net oriented object detection (OOD) model, validating the obtained crystal counts and two-dimensional crystal shape distributions by numerical simulations [28, 29]. The referred to approaches utilizing machine learning often investigate these techniques for model systems under imaging conditions that are not realistic for protein crystallization. In addition to widely varying crystal shapes and sizes, protein crystallization processes are often confronted by additional disturbances resulting from precipitation, aggregation, or thin-film effects. This work studies the possibility of crystal detection models that are effective for a wide range of crystal systems. However, advancing machine learning-based crystal detection for biotechnological applications is limited: robust models obtained through supervised machine learning tasks require large-scale and high-quality data sets. Such data sets are usually obtained in large projects involving extensive manual labeling. However, human labeling of dense systems of almost transparent crystals is considered to be highly error-prone, especially considering that widely used data sets contain annotation errors and biases even in comparably simple annotation tasks [30]. Recent work in the field of facial recognition has demonstrated, that the generation of synthetic data through computer graphics is faster, more cost-efficient, and can avoid the mentioned annotation errors [31, 32]. For microscopy, parametric models of materials science phenomena were used in combination with Perlin noise to generate synthetic images. The parameters of the researchers’ convolutional neural networks were initialized from pre-trained ImageNet models and achieved better segmentation and classification results when trained on their large synthetic data set compared to models fine-tuned on a limited amount of real images [33, 34]. On the other hand, the use of ImageNet pre-training was recently re-examined, and compared to random parameter initialization for general object detection tasks. It was observed that localization-sensitive tasks might benefit from random initialization, provided that large-scale data sets are available [35]. To model the glass-like appearance of crystals under bright-field microscopy using 3D materials and ray tracing algorithms with minimal effort offers the opportunity of transferring this strategy to crystallization processes. The present work is focused on the targeted design of a synthetic data set with the aim of enabling training of robust object detection models, specifically for the quantification and characterization of protein crystallization processes under various and difficult experimental conditions, and specialized in the lower resolution limits of the imaging equipment. First, a synthetic large-scale data set containing 332,558 images of protein crystals in suspension (PCS) for general crystal detection tasks is formulated and generated. All crystals in the PCS data set are fully labeled with oriented bounding boxes, as well as their segmentation masks. Second, it is demonstrated that the PCS data set enables the training of the previously used state-of-the-art OOD crystal detection model from random initialization, resulting in significant improvements of model precision compared to previously used fine-tuning approaches. Finally, the best performing model is used to monitor crystal growth during protein crystallization experiments, validated by measurements of soluble protein concentration of the supernatant of the crystal suspension.

## Materials and methods

### Data set generation

A statistical model is defined, that guides the generation of an unbiased synthetic data set, designed for modern object detection algorithms that leverage deep learning. The set of possible observations represent general minimum area rectangles in image space enclosing crystal objects:

1 $$\begin{aligned} \{r, a, \theta \} \end{aligned}$$

**Fig. 1:**
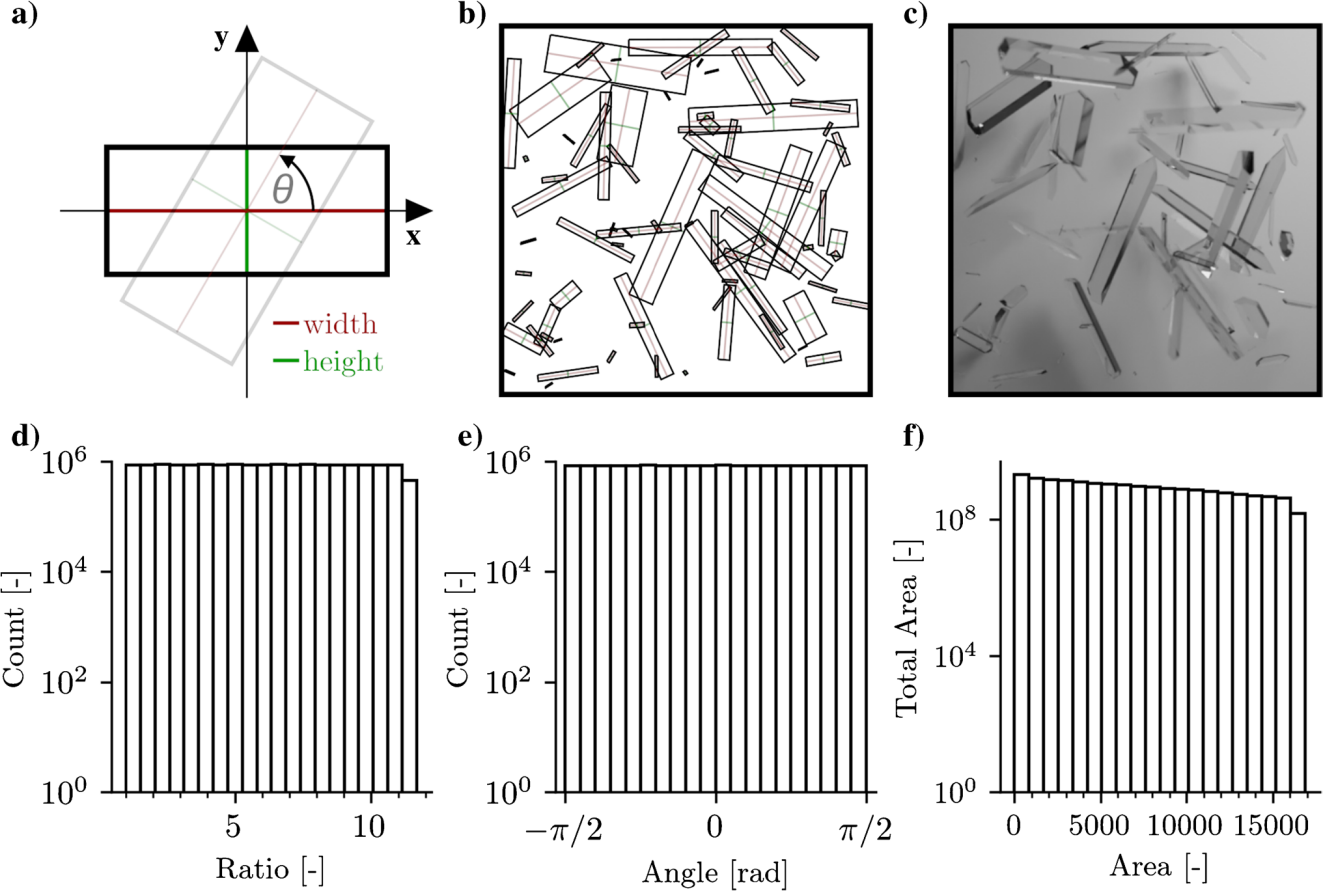
With the statistical model for (**a**) the ratio $r=w/h$, area $a=wh$, and angle $\theta$, virtual scenes (**b**) are generated. (**c**) Example image after crystal placement and deformation, material assignment, randomized placement of the light source, and ray tracing. The crystal counts for different ratios (**d**) and angles (**e**) show good agreement with uniform distributions. (**f**) The total occupied area in each crystal size class decreases slightly for larger objects

Here, *r* is the aspect ratio of the rotated rectangle, *a* its area, and $\theta$ the angle as shown in Fig. 1. The set of probability density functions corresponding to the set of observations is chosen to result in an unbiased data set. This can generally be modelled through continuous uniform distributions $\mathcal {U}_{[a, b]}$ where *a* and *b* define the supported interval:

2 $$\begin{aligned} r\sim & {} \mathcal {U}_{[1, r_{\max }]}\nonumber \\ \theta\sim & {} \mathcal {U}_{[-\pi /2, \pi /2]} \end{aligned}$$

with the maximum aspect ratio set to $r_{\max }=11.4$, approximated by crystals observed during previous experiments. However, a uniform distribution for the area $a \sim \mathcal {U}_{[a_{\min }, a_{\max }]}$ of rotated rectangles results in visually underrepresented smaller crystals, an effect exacerbated by subsequent data augmentation, overlapping crystals, as well as the almost completely transparent appearance of crystals for the common bright-field illumination technique. At the same time, high performance of trained detectors at the lower resolution limit is desired, to yield reliable information about crystal growth during early stages of crystallization processes. This visual underrepresentation of smaller crystals is countered by sampling smaller crystals more frequently, which can be achieved in various ways. Here, constant expected area for infinitesimal intervals is stipulated:

3 $$\begin{aligned} a p(a) da = \mathrm {const.} \end{aligned}$$

which leads to simple reciprocal distribution

4 $$\begin{aligned} p(a) = \left( a\ln \left[ {a_{\max }/a_{\min }}\right] \right) ^{-1} \end{aligned}$$

that causes arbitrarily small crystal size classes to contribute approximately an equal amount to the total crystal area in the data set images. For every sample $(r, a, \theta )$, a random 3D crystal model is picked from a collection of 1851 models. Expressed in homogeneous coordinates, the linear transformation that maps a vertex of the 3D model onto the 2D image plane can be formulated in terms of a uniform scaling matrix $\varvec{S}$, axis rotations $\varvec{R}_z \varvec{R}_y \varvec{R}_x$, translation $\varvec{T}$, camera transform $\varvec{C}$, and a perspective projection matrix $\varvec{P}$:

5 $$\begin{aligned} \varvec{M} = \varvec{P}\varvec{C}\varvec{T} \varvec{R}_z(\gamma ) \varvec{R}_y(\beta ) \varvec{R}_x(\alpha ) \varvec{S}(s_x, s_y, s_z) \end{aligned}$$

The set of 2D points produced by applying this transformation to all vertices of the 3D crystal model is then converted to a rotated rectangle $(r^{\prime }, a^{\prime }, \theta ^{\prime })$ by calculating the convex hull followed by rotating calipers [36, 37]. The transformed 3D model is considered for placement into the virtual scene (Fig. 1) once its projected rotated rectangle $(r^{\prime }, a^{\prime }, \theta ^{\prime })$ is sufficiently close to the sample $(r, a, \theta )$

6 $$\begin{aligned} |\sqrt{a^{\prime }}-\sqrt{a} |< & {} \Delta _a \nonumber \\ |r^{\prime }-r |< & {} \Delta _r \nonumber \\ |\theta ^{\prime }-\theta |< & {} \Delta _\theta \end{aligned}$$

so that finding the correct model transformation $\varvec{M}$ is formulated as an optimization problem. Since the dominant principal axis of 3D crystal models is initially oriented in the $\varvec{\hat{e}}_x$ direction and the extend in $\varvec{\hat{e}}_y$ and $\varvec{\hat{e}}_z$ is approximately equal, $\alpha \sim \mathcal {U}_{[0, 2\pi ]}$ rotations around $\varvec{\hat{e}}_x$ do not affect the optimization process significantly. In contrast, rotations around $\varvec{R}_z(\gamma )$ approximate rotations of $\theta '$. For this to hold, rotations $\beta \sim \mathcal {U}_{[-\pi /3, \pi /3]}$ around $\varvec{\hat{e}}_y$ need to be confined, to not disturb the relation between $\theta '$ and $\gamma$ rotations. Similar considerations lead to the association of scaling $s_x$ and the width $w^{\prime }=\sqrt{a^{\prime }r^{\prime }}$, as well as $s_y$, $s_z$ and height $h^{\prime }=\sqrt{a^{\prime }/r^{\prime }}$. With these approximations, an initial guess for the optimization problem can be formulated:

7 $$\begin{aligned} \gamma _0 (\theta )= & {} \theta \nonumber \\ s_{x,0} (a, r)= & {} c\sqrt{ar}\nonumber \\ s_{y,0} (a, r)= & {} c\sqrt{a/r} \end{aligned}$$

where *c* is an appropriate factor that is calculated after the initial translation $\varvec{T}$. Therefore, the absolute deviations from Eq. 6 can be minimized over the simplified projection matrix

8 $$\begin{aligned} \varvec{M}(\gamma , s_x, s_y) = \varvec{P}\varvec{C}\varvec{T}\varvec{R}_z(\gamma ) \varvec{R}_y \varvec{R}_x \varvec{S}(s_x, s_y) \end{aligned}$$

The maximum allowed absolute deviations during minimization need to be optimized in terms of how close the statistical model should be to the final distributions of the data set (Fig. 1), as well as in terms of computational performance. In this work, it was found that absolute deviations below $\Delta _r < 0.2$, $\Delta _a < 2$, and $\Delta _\theta < {2}^{\circ }$ are sufficient during data set generation. Additional checks are performed for each crystal, so that they do not intersect with the image borders, as well as a maximum overlap constraint with other crystal objects. Once the 3D crystal models are placed in the virtual scene, a glass material with varying index of refraction $n \sim \mathcal {U}_{[1.1, 1.8]}$ is assigned to them. Placement of a light source and subsequent rendering through ray tracing results in photorealistic base images that are used as inputs for the designed data augmentation pipeline. Since crystals are mainly detectable through intensity gradients at their visible edges, limiting the data set to grayscale images enabled a reduction of the complexity of the modelling process while retaining the required information for the crystal detection task. The virtual scenes and images were created with the 3D modelling software Blender [38].

### Data augmentation

Variations of data augmentation are often used in machine learning applications to improve the performance of trained models on inputs that are outside of the sample distributions of the training data set, an effect that is referred to as overfitting and usually caused by biases during the data acquisition process and/or an insufficient size of the training data set. In addition to the design of crystal shape distributions, synthetic noise is modelled such that detectors trained on the PCS data sets are able to perform under a wide range of experimental conditions. This necessarily implies the use of brightness and contrast augmentations, as well as additive gaussian noise. While the former are common augmentations for computer vision applications, the latter is an artifact of imaging techniques. However, these augmentations act either on a local or global scale of the input images, whereas experimental noise can be spatially correlated, such as visible precipitative aggregation, or brightness gradients caused by thin-film interference or skin layers formed by aggregate and/or denatured protein [39]. The PCS augmentation pipeline unifies the treatment of both effects, differing in the underlying generator *N*(*k*) that computes randomized noise textures with a spatial scale of *k*, ranging from a few pixels to a diagonal spanning the image dimensions. While the distinctive cloudy appearance of aggregate is generated by a superposition of Perlin noise [33] textures $N_\mathrm {P}(k)$, thin-film interference or skin formation are approximated through superposition of plane-waves propagating in random directions $\varvec{n}$ with phase shift $\phi \sim \mathcal {U}_{[0, 2\pi ]}$

9 $$\begin{aligned} N_\mathrm {I}(\varvec{x}, \varvec{n}, k) = \frac{1}{2} \left[ \cos \left( 2\pi k^{-1} \varvec{x} \varvec{n} + \phi \right) + 1\right] \end{aligned}$$

where $\varvec{x}$ stands for any image pixel coordinate. Letting $N_{\mathrm {I}/\mathrm {P}}(k)$ denote either of the two noise generators, a noise layer $L_{\mathrm {I}/\mathrm {P}}$ is obtained by averaging $N \sim \mathcal {U}_{\{1, 10\}}$ noise textures with possibly different spatial scales $k_i$

10 $$\begin{aligned} L_{\mathrm {I}/\mathrm {P}} = \frac{1}{N} \sum \limits ^{N}_{i=1} H\left[ N_{\mathrm {I}/\mathrm {P}}(k_i)\right] \end{aligned}$$

**Fig. 2:**
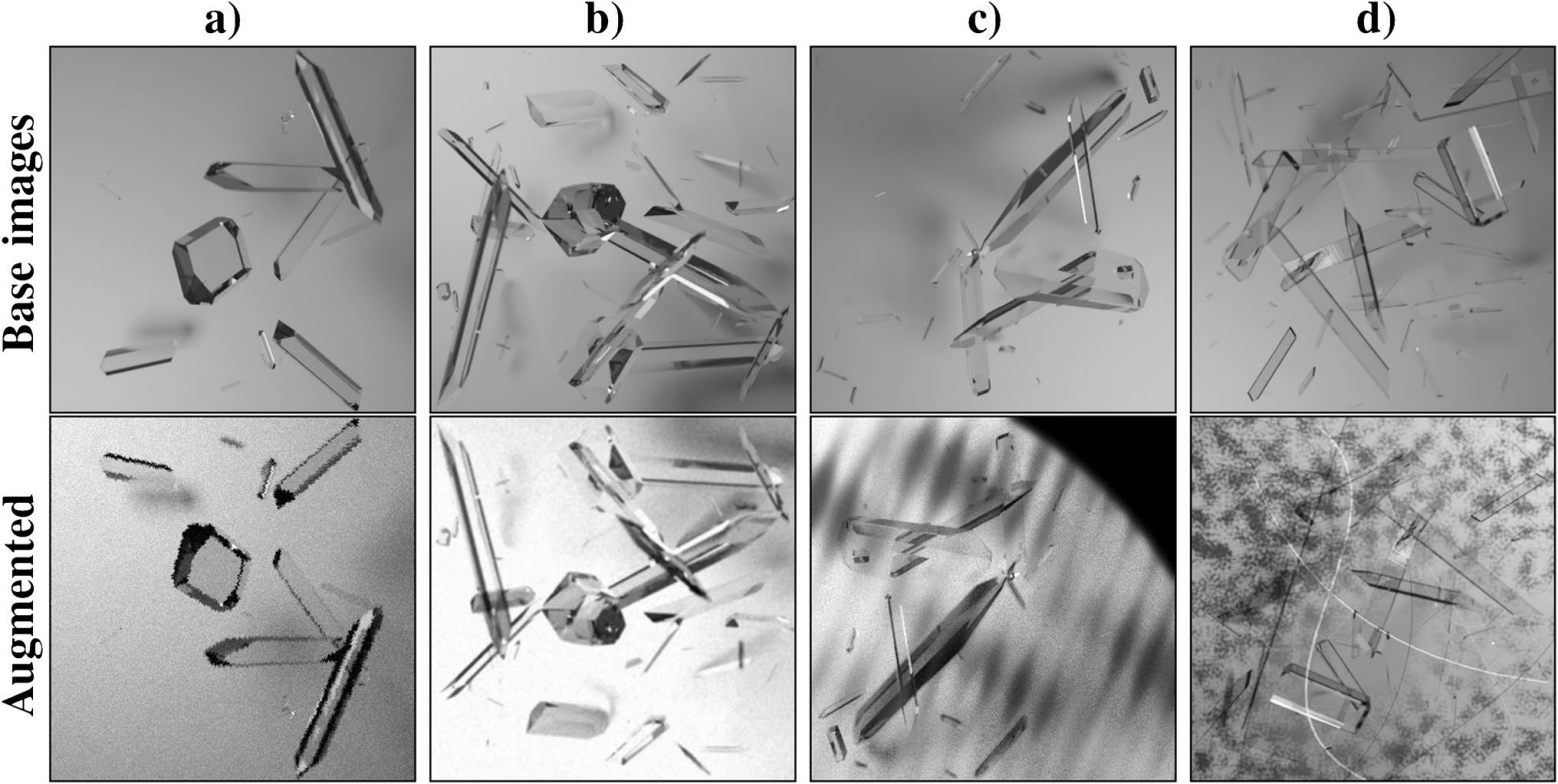
Data augmentations for the PCS data sets visualized. Synthetic base images can be seen in the top row, while augmented images are visible in the bottom row. Examples are for (**a**) edges distortions, (**b**) brightness/contrast variations and blurring (**c**) thin-film interference patterns, per-object highlights, rounded corner overlay, and (**d**) Perlin noise and random splines

To achieve consistent behavior, the histogram equalization operation *H* is introduced, which distributes the intensities of the noise textures evenly. Equipped with noise layers $L_{\mathrm {I}/\mathrm {P}}$, the remaining task is to select an appropriate blending of both images that does not impair the ability of the detector to extract the required information, as is the case if they are simply averaged. Instead, blending of noise layers and input images while maintaining a reasonable signal-to-noise ratio can be achieved more consistently by a weighted average

11 $$\begin{aligned} A(b, l, w_l) = \frac{w_b b + w_l l}{w_b + w_l} \end{aligned}$$

for which a default weight of the input image $w_b=1$ is chosen, such that only appropriate weights for the noise layers need to be determined. First, the noise layer weights are set to a second noise layer $l_{\alpha }$ of the same kind, therefore acting as a randomized transparency layer. Second, noise layer weights are multiplied by the standard deviation $\sigma _b$ of the input image intensities to not overwhelm already weak signals. Lastly, by observing that both types of noise primarily result in dark patterns propagating over the image, noise layer weights are additionally suppressed for high intensity values down to a factor of 1/2 and reinforced up to a factor of 2 for low intensity values, resulting in the final weights

12 $$\begin{aligned} w_l = c \sigma _b l_\alpha 2^{-2l+1} \end{aligned}$$

where *c* is a constant factor acting as a way of tuning the contrast of the noise in the final image. For the PCS data sets, $c=5$ during online augmentation and the generation of the augmented validation data sets. In addition to local or spatially correlated noise layers, object-level augmentations are utilized that brighten or darken single crystal objects slightly relative to their environment, or alternatively faintly outline their refractive borders. Furthermore, random cubic-splines are drawn over the image and might represent scratches or other foreign residues. Similarly, it is possible to round of the corners of the image to simulate images taken from round reactor volumes. Finally, edges of crystal objects can be distorted by displacing entire rows or columns of pixels. A detailed overview of the complete data augmentation pipeline in the correct order can be found in the supplementary information (Table S1 and Figure S1), while Fig. 2 shows examples of augmented base images.

### Machine learning

The one-stage state-of-the-art S$^{2}$A-Net OOD model was used as the basis for all fine-tuning or trained from random initialization experiments, with a ResNet-50 backbone and feature pyramid network (PyTorch 1.9). To facilitate the detection of small and densely packed crystals, the following modifications were introduced. First, two different anchor box configurations were tested and compared. However, since the S$^{2}$A-Net has an anchor refinement network built-in, a positive effect of this modification needs to be demonstrated. Therefore, fine-tuning experiments were carried out with anchor sizes [8, 16, 32, 64, 128] and [16, 32, 64, 128, 256]. Second, the fine details of crystal edges in the input images are crucial information for the detectability of a crystal but might be lost during the initial resolution reduction steps in the default S$^{2}$A-Net model. A model configuration that skips the max-pooling layer after the first convolution was examined, therefore increasing the resolution and information that the following ResNet-50 backbone of the model has access to. Since this modification increases the inference time, advantages and disadvantages were investigated. During fine-tuning experiments, model parameters were initialized from ImageNet pre-trained models, freezing the first stage of the ResNet-50 model. Furthermore, common fine-tuning schedules were adopted. Therefore, eight passes (or epochs) over the 322,558 images from the training data set with learning rate 0.01 were performed, then three passes with learning rate 0.001 and a final pass with learning rate 0.0001. Long training schedules were tried for one ImageNet pre-trained model, as well as one using Kaiming initialization [40]. Both were trained with a learning rate of 0.01 for 80 epochs, then 15 epochs with learning rate 0.001 and final 15 epochs with learning rate 0.0001. All machine learning experiments were conducted on four GPUs (Tesla V100) with two images per GPU. Group normalization was utilized to stabilize training from random initialization [35]. The effects of model modifications were compared on a dedicated validation data set using the common bounding box average precision metric (bbox AP) to compare the performance of different detectors. Average precision is an area-under-the-curve measure of the precision-recall curve that can be extracted from the evaluation of the dedicated evaluation data set. Higher AP scores are interpreted as corresponding to better detectors. To calculate the AP metric, code from the Facebook AI Research Detectron2 project for oriented bounding box evaluation was used [41]. To further elucidate the performance of the detectors for different crystal sizes, the AP metric is separated into three area *a* classes, $\mathrm {AP}_s$ for crystals with their oriented bounding box smaller than $a<8^2$, $\mathrm {AP}_m$ for $8^2<a<24^2$ and $\mathrm {AP}_l$ for $24^2<a$ crystals.

### Crystallization and imaging

Crystals of the model protein *Lactobacillus kefir* alcohol dehydrogenase (*Lk*ADH) were used for image detection to evaluate the trained model by monitoring crystal growth during stirred batch protein crystallization. *Lk*ADH mutants were used to compare the image detection results between similar protein variants. Therefore, *Lk*ADH wild type was mutated by QuikChange site-directed mutagenesis with partial overlapping primers according to Walla et al. (2021) [5] to generate the mutants T102E, Q126K (primers for both mutants recently published [5]) and Q126H (5$'$-GGTATTCACCGTATGAAAAACAAAGG-3$'$ and 5$'$-CATACGGTGAATACCCAGAC-3$'$). *E. coli* BL21(DE3) were transformed with the selected *Lk*ADH variants and produced in shake flasks according to Grob et al. (2020) [4]. After cell lysis by ultrasound and centrifugation of the harvested *E. coli* cell pellets (12000g, 4$^{\circ }$C, 1 h), the supernatant of the *E. coli* cell lysate was dialyzed (20$\upmu$M HEPES/NaOH pH 7.0, 1$\upmu$M MgCl$_{2}$) and filtrated (0.2$\upmu$m polypropylene syringe filter). Stirred batch crystallization (V = 5ml, n$_{\mathrm {stirrer}}$ = 150 rpm) was initiated by addition of equal amounts of precipitation agent (0.1M Tris/HCl pH 7.0, 50 $\upmu$M MgCl$_{2}$, 200 gl$^{-1}$ PEG MME 550) to clarified and dialyzed *E. coli* cell lysate, laid out in the crystallizer located in a temperature controlled water bath (20 $^{\circ }$C) according to Walla et al. (2021) [5]. For protein analysis and image generation, samples were taken at regular intervals for 32 h. To prevent further crystallization, the samples were diluted 10-fold with buffer (20 $\upmu$M HEPES/NaOH pH 7.0, 1mM MgCl$_{2}$). A 10 $\upmu$l droplet was placed in a crystallization plate (MRC UnderOil Crystallization Plate, SWISSCI, Neuheim, Switzerland) and placed under a light microscope (Nikon Eclipse 50i with 4-fold objective (CFI Plan Fluor), Nikon, Düsseldorf, Germany)) in a temperature controlled incubator (20 $^{\circ }$C) for photomicrograph recording. Photomicrographs were taken automatically by a digital camera (DS-Fi3, 0.84$\upmu$m per pixel at 2880 $\times$ 2048 pixel) attached to the microscope, which is controlled by NIS Elements AR v.5.02 imaging software (Nikon, Düsseldorf, Germany). The protein concentration of the crystallization suspension was determined by bicinchoninic acid (BCA) protein assay (Pierce$^{\mathrm {TM}}$ BCA Protein Assay Kit, ThermoFisher Scientific, Munich, Germany) performed with the diluted supernatant of centrifuged samples (13000 g, 20 $^\circ$C, 10 min).

## Results and discussion

### Data set generation and machine learning

A synthetic data set containing 332,558 images of resolution 384 $\times$ 384 pixels was successfully generated using ray tracing algorithms. In combination with specialized data augmentations (see Table S1), highly varying photorealistic scenes of crystallization experiments can be used during machine learning approaches. The data set was split into a training data set containing 322,558 images and 16,801,555 crystal instances, as well as a validation data set containing 10,000 images and 513,337 crystal instances. Figure 1 shows that the size and aspect ratios of the crystals are distributed according to the formulated statistical model $(\mathcal {S}, \mathcal {P})$, therefore guaranteeing an unbiased data set. This is demonstrated through almost constant crystal counts for all histogram bins in the supported intervals of the crystal ratios, angles, and the total crystal area in a corresponding crystal size class. While the latter displays small deviations from the constant behavior, this effect can be explained by the limited image area coupled with a maximum overlap constraint. During training, the specially designed data augmentations modify the base images of the PCS data sets before they are used as input to the model. Additionally, an augmented version of the validation data set was created to enable comparability for future work. Augmented example images are shown in Fig. 2, while Table S1 lists all augmentations applied in the correct order, including flips and rotations as geometric transformations. Different variations of the S$^{2}$A-Net OOD model were trained using either fine-tuning approaches, or starting from random initialization of the model weights. The AP metric is compared for the different detectors in Table 1. Decreasing the size of the smallest anchor from 16 to 8 pixels resulted in better detector performance for small crystals. Furthermore, skipping the max-pooling layer after the first convolutional layer is shown to increase the model performance for all crystal sizes. Additionally, the large-scale PCS data set enabled training procedures that rely on much larger data sets when compared to fine-tuning approaches. Longer training schedules are shown to result in further increases in AP scores. Finally, training object detection models from random initialization using group normalization (GN), results in the highest AP scores of all trained models.

**Table 1:** Comparison of the bounding box average precision metric (bbox AP) for different detector variations based on the $\mathrm {S}^{2}$A-Net model. AP values are further divided into $\mathrm {AP}_s$ for small objects, $\mathrm {AP}_m$ for medium-sized objects and $\mathrm {AP}_l$ for large objects. Evaluation is performed on the PCS validation set. AP values in brackets were evaluated on the augmented PCS validation data set. Higher AP values indicate better crystal detection performance

Anchors	Pool	Initialization	Epochs	AP	$\mathrm {AP}_s$	$\mathrm {AP}_m$	$\mathrm {AP}_l$
16 - 256	−	ImageNet	12	33.48 (18.44)	7.26 (2.16)	34.29 (15.98)	62.75 (42.42)
8 - 128	−	ImageNet	12	34.05 (18.10)	8.80 (2.31)	35.81 (16.10)	60.19 (40.39)
8 - 128	skip	ImageNet	12	38.04 (21.93)	10.91 (3.48)	39.66 (20.69)	65.39 (46.66)
8 - 128	skip	ImageNet	110	58.24 (29.46)	39.00 (8.76)	64.16 (29.19)	76.59 (56.82)
8 - 128	skip	Random	110	60.22 (33.53)	41.87 (12.22)	65.90 (35.35)	77.80 (60.12)

### Experimental validation

**Fig. 3:**
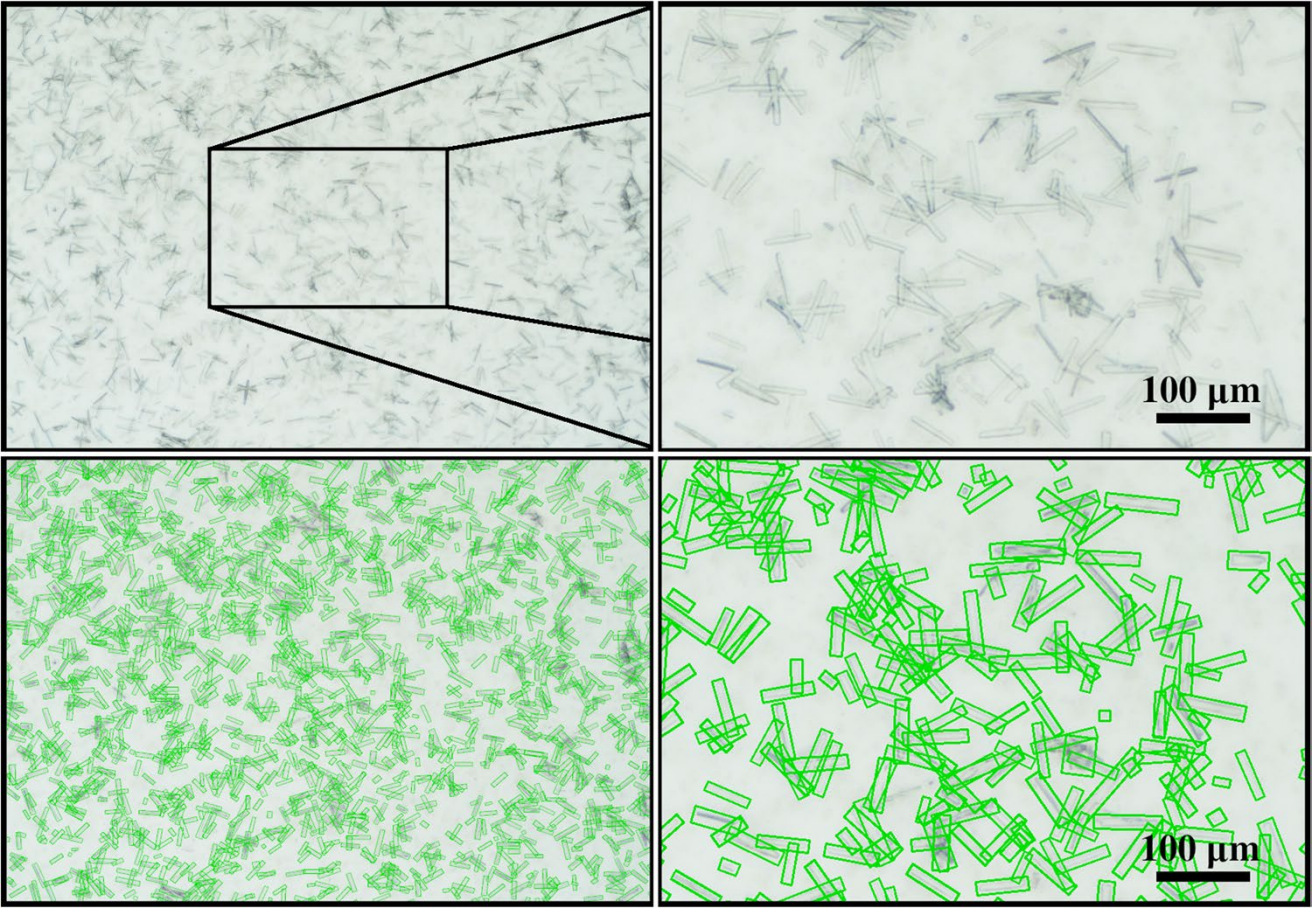
This photomicrography of a diluted 10$\upmu$l droplet sample from a *Lk*ADH crystallization process was taken after 7 h. The width and height of the predicted oriented bounding boxes (green) have been enlarged by six pixels for illustration purposes. In photomicrographs of diluted samples, almost all crystals are detected

**Fig. 4:**
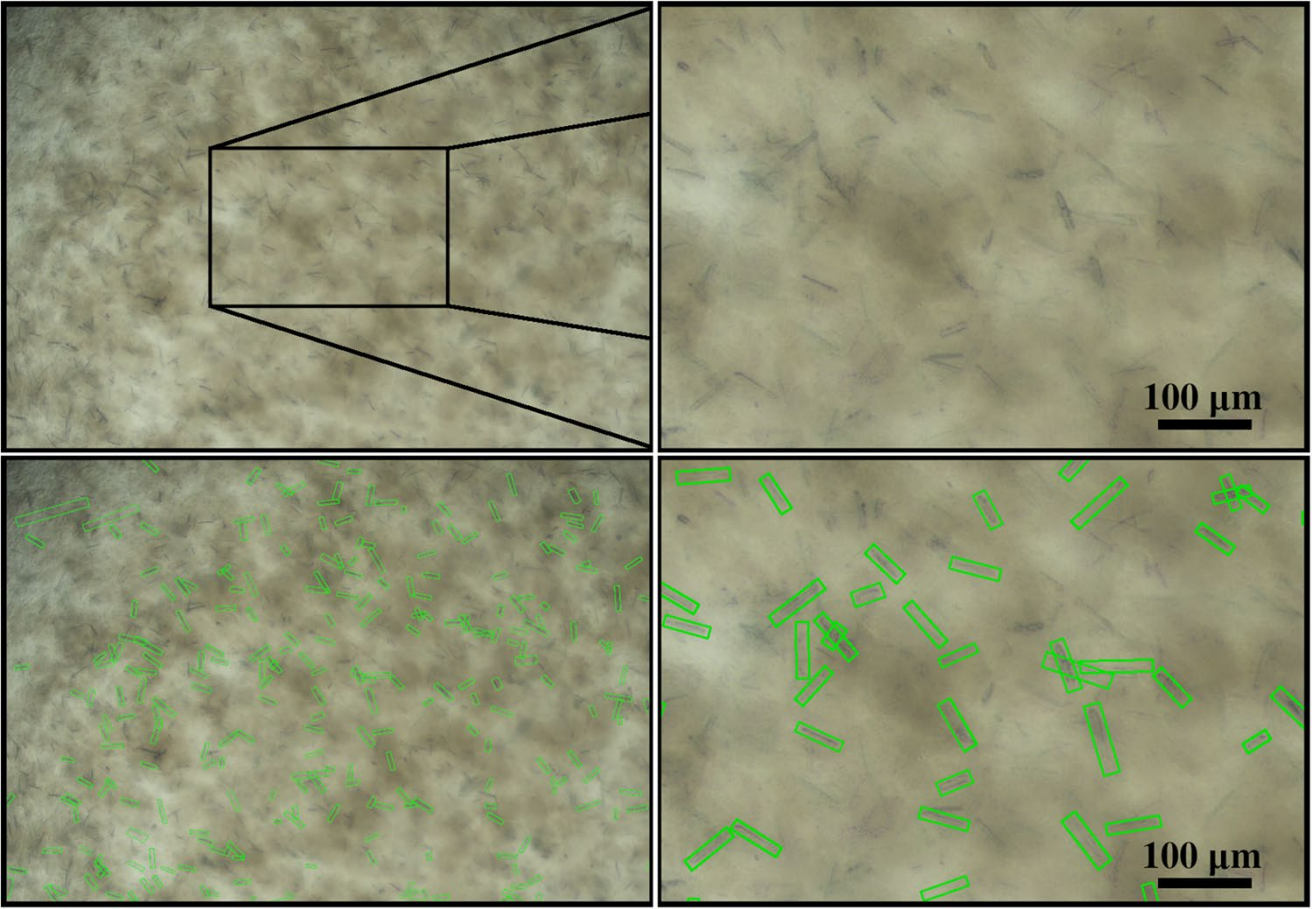
This photomicrography of an undiluted 10$\upmu$l droplet sample from a *Lk*ADH crystallization process was taken after 7 h, depicting the performance of the trained S$^{2}$A-Net model in the presence of noise due to precipitation and/or aggregation. The width and height of the predicted oriented bounding boxes (green) have been enlarged by six pixels for illustration purposes

**Fig. 5:**
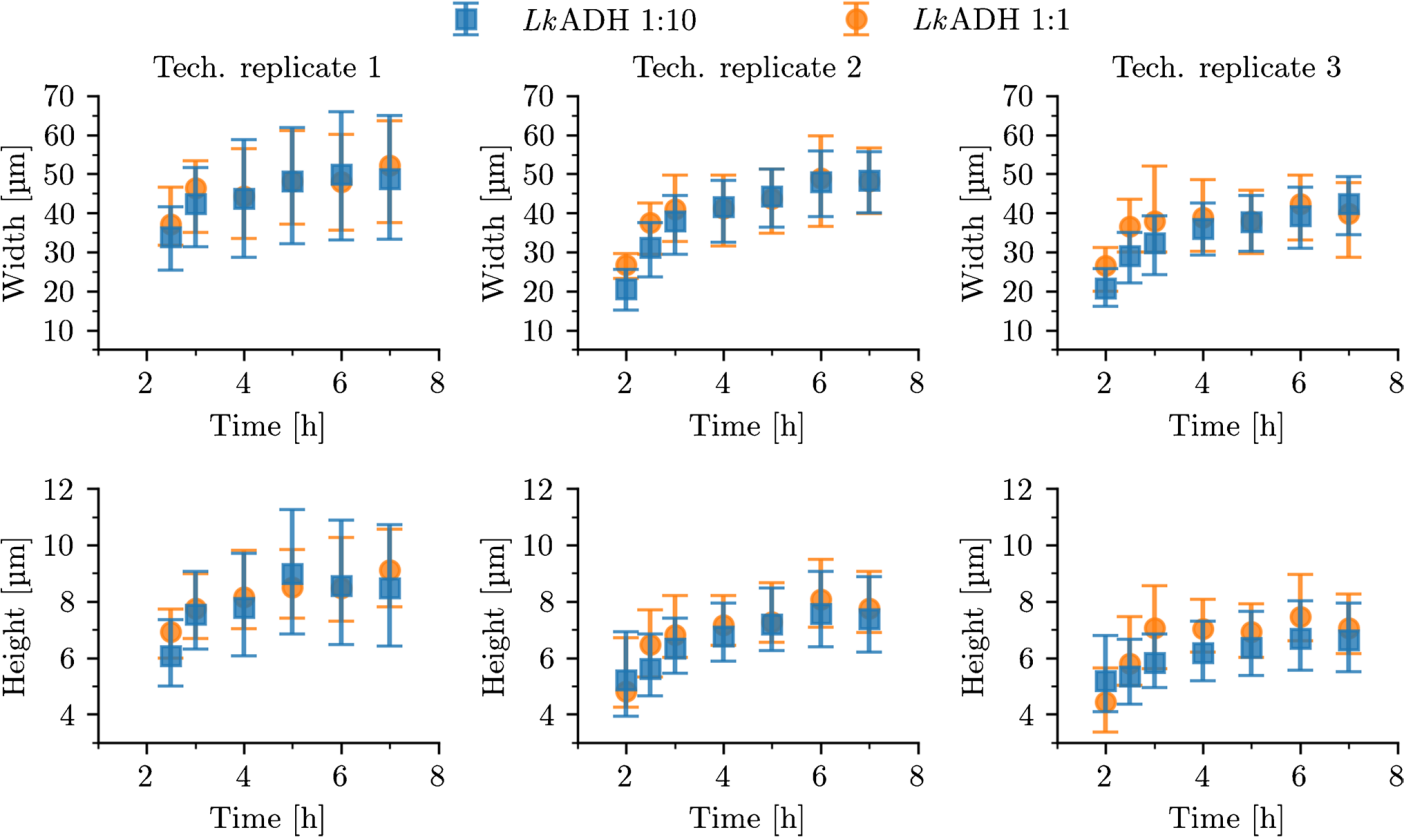
Crystal width and height for three technical replicates of *Lk*ADH wild type batch crystallization experiments in parallel stirred tank reactors (column-wise). Shown are the medians, as well as lower and upper quartiles represented as vertical bars at different times

The model performance has, thus far, only been tested using the synthetic validation data set with the same underlying feature distribution as that of the training data set. While it was previously demonstrated that the PCS data sets are unbiased regarding crystal sizes and shapes, the presented approach to model synthetic noise, lighting conditions, contrast variations, and aspects of the observed reactor volume requires evaluation on real-world data. Multiple batch crystallization experiments were conducted to investigate the performance transfer from synthetic data to real data. All images were evaluated with the best performing S$^{2}$A-Net model from Table 1 which was trained from random initialization using the synthetic PCS data set. Automated preprocessing steps first converted photomicrographs to grayscale images. Additionally, grayscale intensity distributions of each photomicrograph were shifted to the distribution used during training. To test the robustness of this model, three technically replicated protein crystallization processes were conducted. High-resolution photomicrographs with 2272 $\times$ 1632 resolution were taken at different times to capture the crystal growth process of the selected model protein *Lk*ADH. While diluted samples result in clear images (Fig. 3), undiluted samples are subject to noise by precipitation and/or aggregation due to the unprocessed sample containing high amounts of host cell protein (Fig. 4). While the modified S$^{2}$A-Net model detects less crystals than in the undiluted case, the extracted crystal size distributions can be demonstrated to be similar. Figure 5 compares the resulting medians of the widths and heights of the detected crystal objects in 10 $\upmu$l droplets which were sampled at different times during the process. While distributions from diluted and undiluted images generally align well, deviations lead to a slight overestimation of the widths or heights for the undiluted samples at the earlier times during the crystallization process. Here, the size of crystals is on the scale of a few image pixels (${0.84}~{\upmu }\text {m}/\text {px}$) and such deviations can therefore be attributed to an inherent limitation due to the imaging equipment. The robustness to noise of the trained S$^{2}$A-Net model is further demonstrated through multiple two-sample Kolmogorov-Smirnov tests [42], which are unable to reject the hypothesis that the widths of detected crystals from the diluted and undiluted photomicrographs are drawn from the same distribution (Table 2). Here, in addition to comparing the distributions shapes, the numbers of detected crystals from diluted and undiluted samples are provided. The detected number of crystals steadily increases for diluted and undiluted samples during the early stages of the crystallization process. However, at later times the crystal count is subject to fluctuations especially for the undiluted noisy samples. This might be caused by the manual sampling process, which can be improved upon by further automation and online microscopy probes. Furthermore, different equilibrium crystal size distributions indicate that stirred crystallization experiments are subject to small variabilities even when started from the same biological batch.

**Table 2:** Two-sided two-sample Kolmogorov-Smirnov test for the hypothesis that the crystal width distributions from diluted and undiluted samples in a technical replicate of *Lk*ADH wild type stirred tank crystallization are identical at the sampled time points. Bold p-values indicate the failure of the test to reject the hypothesis for a significance level of 5% in favor of the alternative that the distributions are different. Additionally, the table contains information about the number of detected crystals of diluted $\mathrm {N_d}$ and undiluted samples $\mathrm {N}$

	Techn. Replicate 1			Techn. Replicate 2			Techn. Replicate 3		
t [h]	p-value	$\mathrm {N_d}$	N	p-value	$\mathrm {N_d}$	N	p-value	$\mathrm {N_d}$	N
2.0	**0.27**	26	6	**0.41**	108	26	**0.32**	173	22
2.5	**0.19**	153	73	**0.26**	546	83	**0.37**	862	54
3.0	**0.11**	352	253	**0.19**	916	178	**0.29**	1246	104
4.0	**0.13**	496	231	**0.06**	1189	257	**0.16**	1145	150
5.0	**0.08**	406	196	**0.10**	1319	167	**0.08**	1050	89
6.0	**0.13**	241	107	**0.10**	1283	81	**0.12**	989	84
7.0	**0.10**	512	105	**0.06**	1004	124	**0.17**	1451	47

**Fig. 6:**
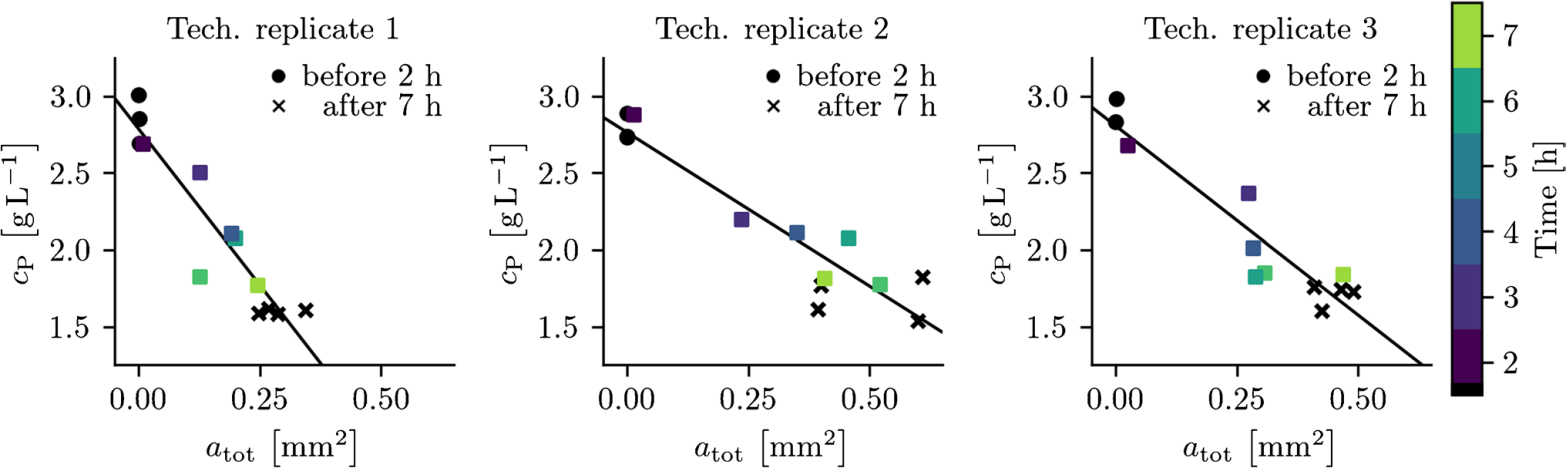
Protein concentration of supernatant ($c_\mathrm {P}$) measured at different times during the *Lk*ADH wild type crystallization process for the three technical replicates correlates with the total crystal area ($a_\mathrm {tot}$) detected by the best performing trained S$^{2}$A-Net model in photomicrographs of the diluted samples

**Fig. 7:**
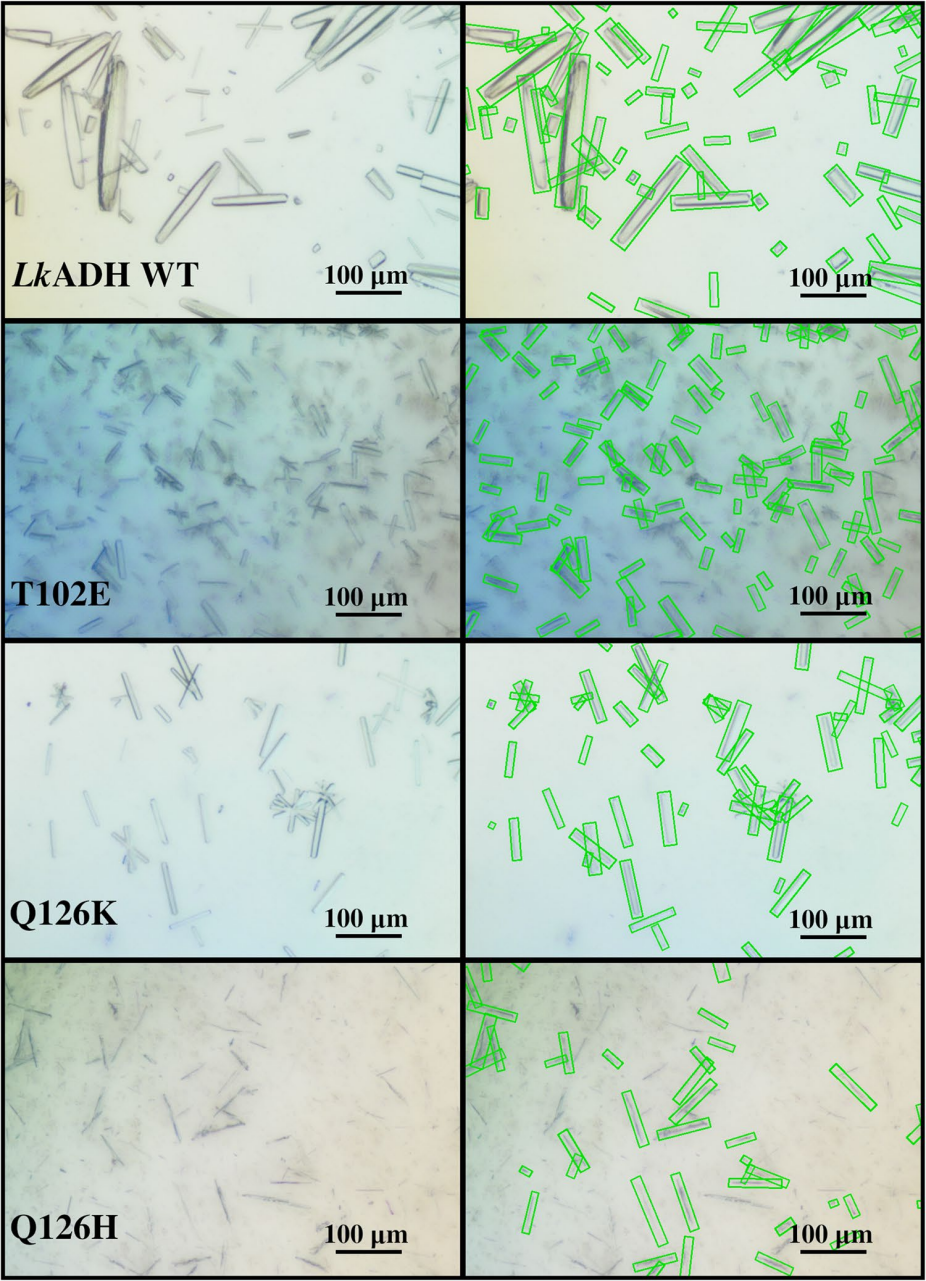
Central cropped sections of photomicrographs of samples taken after 24 h crystallization of *Lk*ADH wild type (WT) and mutants T102E, Q126K, and Q126H (left) with the corresponding predictions of the best performing trained S$^{2}$A-Net model (right). The width and height of the predicted oriented bounding boxes (green) have been enlarged by six pixels for illustration purposes

**Fig. 8:**
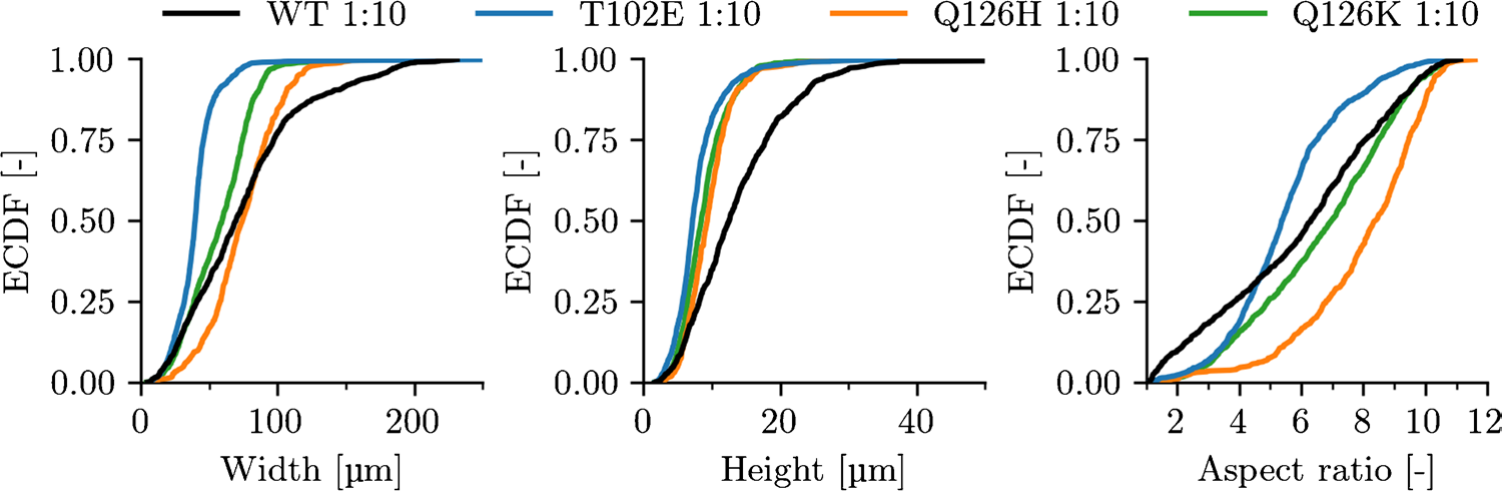
Empirical cumulative distribution functions (ECDF) of the *Lk*ADH wild type (WT) and mutants T102E, Q126K, and Q126H evaluated from photomicrographs taken after 24 h of crystallization in stirred tank reactors. Shown are crystal lengths, widths and aspect ratios extracted from the photomicrographs

Since almost all crystals are detected by the trained S$^{2}$A-Net model in the case of diluted samples, the general question of to what degree the detected crystal growth correlates with the protein concentration of supernatant is addressed in Fig. 6, indicating that both measurements can be described by a linear relationship (see Table S2). However, while technical replicates two and three behave similarly under these metrics, the first technical replicate results in less total crystal area in the analyzed photomicrographs while reaching the same final protein concentration of supernatant. In addition to the previous considerations concerning the model’s robustness to noise, to demonstrate the model’s robustness to varying crystal sizes and aspect ratios, crystallization of four *Lk*ADH variants with different crystallization kinetics was conducted. In prior studies with the homologous *Lactobacillus brevis* ADH (*Lb*ADH), mutants T102E and Q126H resulted in different crystal sizes compared to the *Lb*ADH wild type when crystallized starting from identical initial conditions [4]. Recent work transferred the mutations T102E and Q126K to the *Lk*ADH and demonstrated a transfer of crystallizability for the *Lk*ADH wild type and these mutants [5]. In this study, the transfer to *Lk*ADH was extended to mutant Q126H and the aspect ratio of the *Lk*ADH wild type and mutants was examined in addition to width and height of the crystals during the crystallization process. Central cropped sections of selected representative images can be seen in Fig. 7, while Fig. 8 shows empirical cumulative distribution functions (ECDF) for crystal width, height, and aspect ratio. In previous work, the widths and heights of the *Lk*ADH wild type and mutants T102E and Q126K have been measured manually to analyze crystallization behavior under equal initial protein concentrations [5]. With the presented trained S$^{2}$A-Net model, experiments with the same mutants result in an identical order of crystal sizes for widths and heights: WT > Q126K > T102E. However, while previously the wild type had the highest aspect ratio amongst all *Lk*ADH variants, the current analysis suggests higher aspect ratios for the *Lk*ADH mutant Q126K. It is suspected that the present unbiased S$^{2}$A-Net model is more likely to detect small and more compact crystals than manual labeling. This is supported by the rapidly increasing empirical distribution function of crystal widths for the wild type. Such widely varying crystal sizes are not present for the mutations T102E, Q126K, and Q126H. A possible explanation for this effect might be a tendency of the wild type to break during stirred crystallization experiments, while in contrast, the previous work has demonstrated stronger intermolecular interactions for protein crystals of the mutant T102E [5]. The newly transferred *Lk*ADH mutant Q126H from the *Lb*ADH is larger than the mutant Q126K, but remains smaller than the wild type on average. Moreover, Fig. 8 shows that mutants T102E and Q126H can be clearly distinguished from the *Lk*ADH wild type and mutant Q126K by their aspect ratio. More specifically, mutant T102E results in small and compact crystals with average $r=5.50\pm 1.77$, and mutant Q126H exhibits large average aspect ratios $r=7.97\pm 2.00$, while the wild type with $r=5.99\pm 2.64$ and the mutant Q126K with $r=6.70\pm 2.25$ are positioned between them.

## Conclusions

A large-scale and unbiased data set for supervised learning of crystal detection tasks was generated, thus circumventing the previous error-prone and time-intensive manual labeling task of crystal objects. This large-scale data set enabled the training of state-of-the-art oriented object detection models. By evaluating the performance of different models on a validation data set, it was found that crystal detection tasks profit from smaller anchor sizes, skipping of the initial resolution reduction through max-pooling, and random initialization coupled with long training schedules. The best performing model was validated experimentally, demonstrating its robustness to noise, and varying crystal sizes and aspect ratios. It was also demonstrated that the model is able to detect crystals at the lower resolution limit of the imaging equipment, thus being able to monitor the growth of *Lk*ADH crystals during early stages of the process. Furthermore, differences in crystal shapes of the *Lk*ADH wild type and mutants T102E, Q126K, and Q126H were analyzed and compared to previous manual measurements. Due to the robustness of the model against noise-like aggregation or precipitation, this work has high potential for the monitoring of crystallization using online microscopy probes in technical processes. Finally, due to the flexibility of the synthetic data set generating process, future work might extend it to model and classify different crystal morphologies, crystal clusters, or the effects of phase separation.

## Supplementary Information

Below is the link to the electronic supplementary material.

Supplementary file1 (PDF 747 KB)

## Data Availability

The training data set, validation data set, augmented validation data set, and parameters of the trained modified S$^{2}$A-Net model can be downloaded from the LRZ Sync+Share service via the link: https://syncandshare.lrz.de/getlink/fiQmpeVNi4XKJ9ioGLsTSVJY. Authors can confirm that all relevant data are included in the article and/or can be obtained from the above links.

## Code availability

The generating source code of the PCS data set, as well as the source code for the specialized online augmentations is accessible on GitHub: https://github.com/bisdan/pcs.
