# Supplementary material for: Machine learning-based protein crystal detection for monitoring of crystallization processes enabled with large-scale synthetic data sets of photorealistic images
Source: Anal Bioanal Chem. 2022 Jun 4;414(21):6379–91. doi: 10.1007/s00216-022-04101-8 (PMC9372129; doi:10.1007/s00216-022-04101-8)
Supplement: Supplementary file 1 — Supplementary file1 (PDF 747 KB) [file 216_2022_4101_MOESM1_ESM.pdf]

Supplementary Information

Machine-learning Based Protein Crystal Detection for Monitoring of Crystallization Processes Enabled with Large-scale Synthetic Data Sets of Photorealistic Images

Daniel Bischoff[1*], Brigitte Walla[1] and Dirk Weuster-Botz[1]

[1*]Technical University of Munich, Institute of Biochemical Engineering, Boltzmannstr. 15, Garching, 85748, Bavaria, Germany.

*Corresponding author(s). E-mail(s): daniel.bischoff@tum.de; Contributing authors: brigitte.walla@tum.de; dirk.weuster-botz@tum.de;

Data augmentation pipeline

Table S1 Data augmentation pipeline of the PCS data sets in the order applied during training and creation of the augmented PCS validation data sets, with image probabilities p_i and per-object probabilities p_o.

	Augmentation	Description	p_i	p_o	Blending
1.	Highlight	Modify single crystals	0.5	0.2	additive
2.	Splines	Straight or curved lines	0.2	-	additive
3.	Distort	Displacing pixels	0.05	-	-
4.	Gauss	Gaussian noise	0.4	-	additive
5.	Impulse	Impulse noise	0.2	-	additive
6.	Brightness	Brightness variation	1.0	-	-
7.	Contrast	Contrast variation	1.0	-	-
8.	Waves	Interference patterns	0.4	-	weighted average (Eq. 11)
9.	Perlin	Perlin noise	0.4	-	weighted average (Eq. 11)
10.	Contrast	Contrast variation	1.0	-	-
11.	Overlay	Circular overlay	0.2	-	additive
12.	Blur	Gaussian blurring	0.7	-	-
13.	Rotation	Random 90° rotations	1.0	-	-
14.	Flip	Horizontal and vertical flips	1.0	-	-

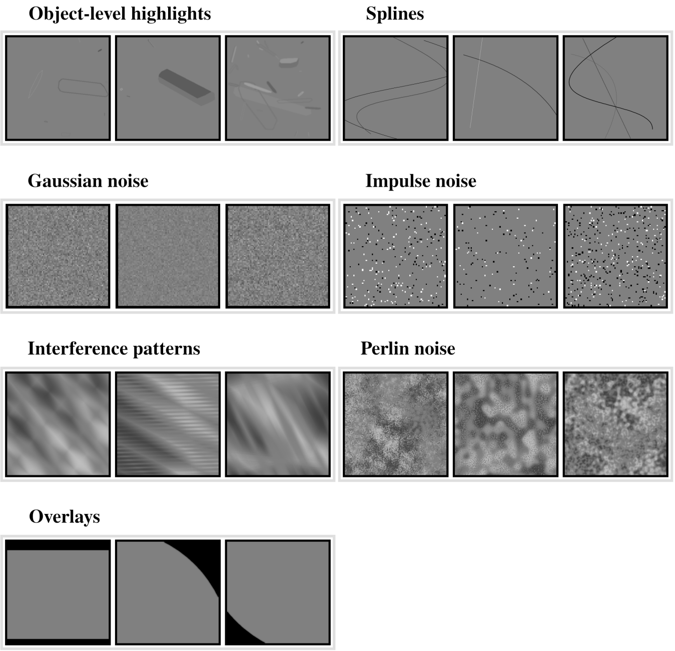

Fig. S1 Example noise layers that are blended with the base images during data augmentation. Augmentations have been applied to a gray image for illustration purposes

Regression results

Table S2 Linear regression results for the relationship of the protein concentration of supernatant (c_P) and the total detected crystal area per image (a_{tot}) of Figure 6. Besides slope and intercept of the linear regression, the standard error (SE) and r-squared value (R^2) are provided.

Tech. replicate	Slope [$g\,l^{-1}\,mm^{-2}$]	Intercept [$g\,l^{-1}$]	SE [$g\,l^{-1}$]	R^2
1	-4.06	2.79	0.47	0.87
2	-2.00	2.76	0.23	0.87
3	-2.46	2.81	0.28	0.88
